# Evaluation of the Effectiveness of Light Streamer Tori-Lines and Characteristics of Bait Attacks by Seabirds in the Western North Pacific

**DOI:** 10.1371/journal.pone.0037546

**Published:** 2012-05-25

**Authors:** Noriyosi Sato, Daisuke Ochi, Hiroshi Minami, Kotaro Yokawa

**Affiliations:** 1 National Research Institute of Far Seas Fisheries, Shimizu, Shizuoka, Japan; 2 Graduate School of Fisheries Science and Environmental Studies, Nagasaki University, Nagasaki, Japan; Institute of Marine Research, Norway

## Abstract

To improve the effectiveness of tori-lines it is necessary to evaluate the ability of tori-lines to mitigate seabird bycatch and determine what kind of seabird species gather during line settings, attack the bait and are incidentally caught. We conducted two experiments in the western North Pacific and examined the effectiveness for seabird mitigation of light streamer tori-lines which have no long streamers but many light (short) streamers and are mainly used in the North Pacific area. Firstly, the effectiveness of two different types of tori-line (light streamer (1 m) and long streamer (up to 7 m) tori-line) and of two different colors (yellow and red) of light streamers for seabird bycatch avoidance was evaluated using 567 sets based on data from 20 offshore surface commercial longliners. No significant difference in the bycatch number between the different tori-line types and streamer colors was found. Secondly, we investigated the characteristics of the seabird bycatch in the North Pacific and the effectiveness of three different types of streamers (light, hybrid and modified light types) by detailed observations of seabird attacks using a chartered longline vessel. Although the appearance rate of albatrosses and shearwaters were 40.9% and 27.7%, Laysan albatross was the main seabird species that followed the vessel but shearwaters seldom followed the vessel and did not aggregate during line setting. In all attacks on bait observed during line settings, 81% and 7% were by albatrosses and shearwaters, respectively. In the number of primary attacks by Laysan albatrosses which attacked most aggressively of all seabirds, there were no significant differences among the tori-line types. No individuals of shearwater were caught. The results of both experiments indicated that light streamer tori-lines were as effective as tori-lines with long streamers for mitigating seabird bycatch in the North Pacific.

## Introduction

Most seabird bycatches in pelagic longline fisheries occur when seabirds attack the bait thrown into the sea before the bait sinks to the fishing depth [1]. Seabird bycatch by tuna longline fisheries has been shown to cause a negative impact on some seabird populations [1], [2], and development of effective mitigation measures is needed which will also have an effect of improving the efficiency of the fishery by reducing bait loss. Seventeen out of the 22 albatross species are ranked in the category of threatened in the 2010 Red List [3], and urgent countermeasures are required.

Tori-lines were developed by Japanese fishermen to reduce the seabird bycatch and reduced bait loss. This mitigation method deters seabirds from approaching the vessels due to the motion of the streamers, and allows the bait to sink to a sufficient depth where seabirds cannot attack the bait [1]. This mitigation measure has a high effectiveness to reduce the bycatch in both pelagic and demersal longline fisheries [1], [2], [4], [5], [6], and is used by many longline vessels as the most practical measure because it is cost effective and it does not require significant changes to the fishing gear or vessel to use it [6], [7].

The effectiveness of tori-lines is affected by various factors such as the weather and the hanging position of the tori-line in relation to the baited hooks [8], [9], [10]. It is considered that the streamers of the tori-line play an important role to intimidate seabirds from coming into close proximity and thereby minimize them accessing the thrown bait. Moreover, the length and material of the streamers would relate to the frequency and ease with which it becomes tangled with the fishing gear. However, there are few studies to investigate the actual streamer effect on seabird bycatch.

In the Western and Central Pacific Fisheries Commission (WCPFC), long line vessels of commission members (which include Japan), cooperating non-members and participating territories are required to use at least two of the 10 mitigation measures [11]. Tori-lines are used by many longliners as the most practical measure of these mitigation measures. From the guidelines of the WCPFC, two types of tori-line have been mainly used in the western North Pacific. One is the long streamer tori-line with dangling long streamers and the other is the short (light) streamer tori-line with short streamers. Previous researche studies conducted in the North Pacific indicated that the light streamer tori-line is effective for reducing the bycatch [12], [13], [14]. Although these results showed the effectiveness was not significantly different between both types of tori-line, the sample size was comparatively small and the research period was limited from April to July because the data were collected by a limited number of research vessels. Melvin et al. [15] compared the effectiveness of a light streamer tori-line with a hybrid streamer tori-line. The hybrid streamer tori-line was configured using both long streamers and short streamers and each type of streamer was used to protect within 50 m and over 50 m from the vessel, respectively. There were no differences in the ability of avoiding seabird access between the light streamer and the hybrid streamer tori-lines for surface foragers like albatross, but the ability of the hybrid streamer tori-line to avoid the access of diving seabirds was higher than the light streamer tori-line, which suggests that the hybrid streamer tori-line was effective in the South African exclusive economic zone (EEZ) [15]. However, the fauna of seabirds varies greatly from region to region and it is unknown whether this hybrid streamer tori-line is best all over the world. To develop a tori-line that fishermen can use easily and reduce seabird bycatch, it is necessary to investigate the effectiveness of various tori-line types and behaviors of seabirds in various regions.

Depending on the species composition of the seabird community, bycatch risk may vary. Seabird species exhibit diverse foraging skills to find and catch prey [16]. Although almost all albatross species have poor diving skills and hence forage on drifting squids and fishes at the sea surface, many shearwater species have an ability to dive deeper than albatrosses and they are able to chase fish during their deeper diving [16], [17]. Recently, Melvin et al. [15] reported that there are two categories of seabird attacks: primary and secondary attack. Primary attack is an attempt by a seabird to take the bait from a hook. Secondary attack is an attack by another bird(s) on the primary bird as the bait is brought to the surface. When many diving seabirds (e.g. petrels, gannets and murres) are crowded around the vessel during a longline operation, their attacks lead to many bycatches of albatrosses which are hooked during the secondary attack [15].

The seabird community in the western North Pacific is constituted of many surface foragers (Laysan albatross (*Phoebastria immutabilis*), black-footed albatross (*P. nigripes*) and short-tailed albatross (*P. albatrus*)) and diving foragers (e.g. streaked shearwater (*Calonectris leucomelas*), flesh-footed shearwater (*Puffinus carneipes*), wedge-tailed shearwater (*P. pacificus*), sooty shearwater (*P. griseus*) and short-tailed shearwater (*P. tenuirostris*)) [17]. Therefore it is necessary to examine whether albatross bycatch in the western North Pacific is primarily due to primary or secondary attacks.

In this paper, two experiments were conducted to investigate the effectiveness for seabird mitigation of light streamer tori-lines which are mainly used in the North Pacific area and develop a more effective tori-line design. In experiment 1, we compared bycatch number between long streamer and light streamer tori-lines using data from 20 offshore commercial longliners. The effectiveness of different colors of streamer (yellow and red) for seabird bycatch was also investigated. In experiment 2, we conducted detailed observations of the bait attacking behavior using a chartered longliner vessel. The attack number are compared among three types of tori-lines; a light streamer, a hybrid streamer and a modified light streamer tori-line devised in this study, and the characteristics of seabird bycatch in the North Pacific were investigated.

## Methods

### Procedure of Experiment 1

**Figure 1:**
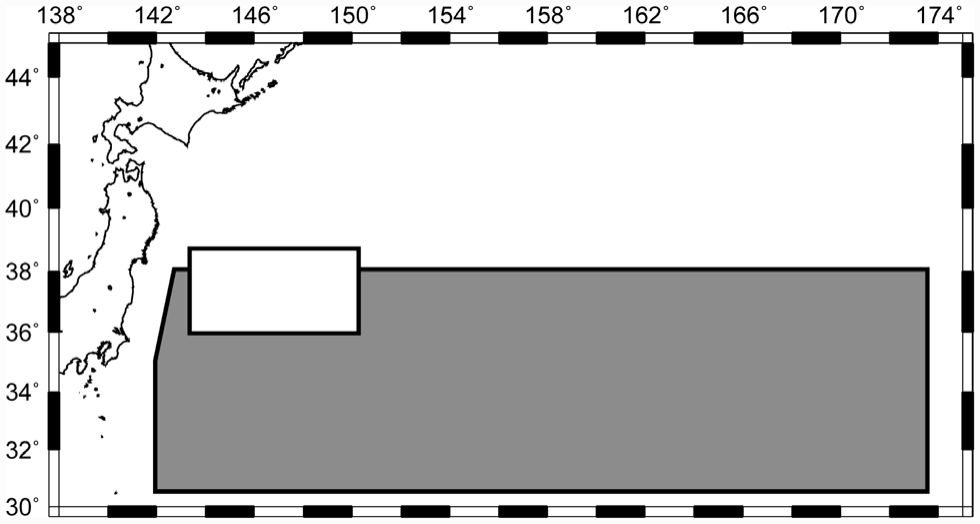
The operational area. Grey and white color zones indicated the operating ranges in experiment 1 and 2, respectively.

This experiment was carried out using the offshore commercial longline fleet, based from Kesennuma fishing port, north-eastern Honshu, Japan. The operational area of this fleet is in the transition zone between the Kuroshio-warm current and the Oyashio-cold current (Figure 1). The research was conducted in the period between January and March 2010 when the number of seabirds appearing in the fishing ground of the Japanese offshore longliners become largest during the year, and 20 longline vessels (over 120 GRT) were engaged in the research. Each longline vessel that participated in this research generally operates 20–60 sets with 2 cruises during the research period. The operations were a night soak style: although most line settings were started in the afternoon and completed around a few hours after sunset, about 10% of all settings were started after sunset. This line setting time is standard practice for the fishery in this area. Fishing gear was the shallow-set style, see below for details. This field research was approved by the “Fisheries Agency of Japan”.

**Figure 2:**
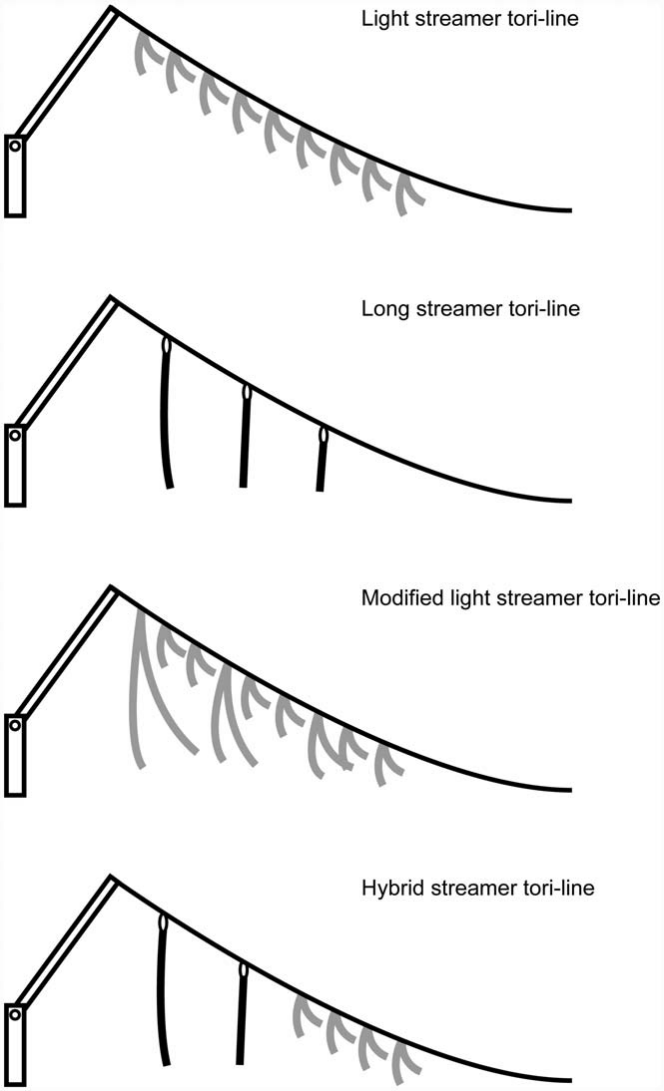
A schematic representation of the four types of tori-line. Grey streamer indicated polypropylene (PP) band spliced into the backbone. Black streamer indicated Nylon cord in long streamer tori-line and UV-coated rubber tube in hybrid streamer tori-line and were clipped to swivels in the backbone.

To test the effectiveness of the two different types of tori-line (light streamer and long streamer tori-line; Figure 2) and two different colors (yellow and red) of short streamers, the period of research was divided into two phases and defined as “phase 1” and “phase 2”. In phase 1, the two types of tori-lines with the different streamer types were deployed during line settings and all 20 longline vessels participated in this phase. During phase 2, two types of light streamer tori-line with different colors (yellow or red) were deployed by 8 vessels. In each phase, the two different designs of tori-lines were alternatively deployed each line setting to arrange the same experimental condition between the two designs of tori-line.

In this study, data collection in experiment 1 was conducted by the fishermen of the offshore commercial longline fleet in Kesennuma. We have conducted research every year from 2008 [18], and explained the importance of this research to them. All skippers fully recognize the significance of our research. They also closely cooperated with this research because bait loss caused by seabird foraging, which subsequently leads to the bycatch is a serious economic problem for them. It is thought that any false reporting was negligible. We judged that the data recorded by fishermen are reliable. In each gear setting, the number of hooks set, largest number of observed seabirds (including non-albatross species) and deployed tori-line types were recorded by the skipper of each vessel. During line hauling observations, the number of seabirds caught was recorded by species during gear hauling, however, albatrosses were the only species observed. All skippers reported that the length of aerial extent in all tori-lines had been maintained at 50–60 m throughout all operations. We eliminated the data for two vessels in the analysis from the original total of 22 and 9 vessels taking part in experiment 1. One vessel used a double tori-line and the other did not consistently record the data. Subsequently, the data of 20 and 8 vessels were analyzed in phase 1 and phase 2, respectively.

### Procedure of Experiment 2

A research vessel, RV “Taikei-maru No. 2” (42.4 m, 196 GRT) was used for the experiments in the western North Pacific, 10 April–7 June 2010. This field research was approved by the “Fisheries Agency of Japan”. The operational area of this vessel is shown in Figure 1. Although some operations were conducted within the Japanese EEZ, the number of seabirds was few. Twenty-four longline operations were carried out. The operation was a night soak style: line setting was started in the afternoon and completed before sunset. Hauling began at dawn. Fishing gear was the shallow-set style and the target fishing depth was 40–70 m (the buoy lines was 8 m). The lengths of branch lines and wire leader were 18 m (15 m plus 1.5 m) and 1.5 m, respectively. Each basket had four hooks and branch lines. We used 240 baskets (960 hooks) per one operation. The bait casting was conducted by the fisherman's throwing. Whole mackerel (*Scomber japonicus*) were used as the fishing bait. Longlines were typically deployed at a 7.5 knot speed over ground.

The tori-lines were attached to the 7.8 m pole made of glass-fiber (about 10 m above the water) installed on the portside of the stern deck. Angle of the pole was adjusted so that the tori-line was located above the sinking baited-hooks. The length of the aerial extent in all tori-lines was maintained at 90–100 m throughout all operations. No offal was discharged during line setting.

One operation was divided into three blocks (one block consisted of 320 hooks), and we used different types of tori-lines (light streamer, hybrid streamer or modified light streamer tori-line; Figure 2) for each block in a fishing operation. This block-designed experiment was expected to cancel the heterogeneity and other random factors affecting the bait-taking behavior of seabirds among the three treatments within and between fishing operations.

During line setting, behavioral observations of seabirds were made by two researchers. The method described by Melvin et al. [15] was introduced for the collection of data of the seabird attacking behaviors. We allocated two 20 min observation sessions (two researchers observed for 20 min alternately) for each block. Each session consisted of two parts, seabird abundance that aggregated within a 250 m hemisphere centered on the stern of the vessel was counted with their species identified during the first 5 min. Then, the next 15 min of the session, the number of attacks on bait was counted by species. We counted only dives and underwater plunges over baited hooks as a primary attack. In all attack behaviors, the primary attacks were counted based on the distance astern (0–25 m, 26–50 m, 51–75 m, 76–100 m, 101–125 m, 126–150 m and 151–200 m) and the location relative to the tori-lines (whether starboard or port of the tori-line). The distance was estimated based on the length of the tori-line. Secondary attacks (other birds fighting for the bait brought to the surface by the bird making the primary attack) were also recorded.

During the gear hauling, the number of seabirds caught in each block was recorded by species.

### Specification of tori-lines compared

Two and three designs were deployed in experiment 1 and 2, respectively (Table 1 and see also Figure 2). All light streamers were polypropylene (PP) band spliced into the backbone of the tori-line and tied at the centre. Modified light streamer tori-line had a long PP band every five streamers and these PP bands were also spliced and tied in the same way as the light streamers. Long streamers of the long streamer tori-line as used in experiment 1 and the hybrid streamer tori-line in experiment 2 were clipped to swivels in the backbone. In the hybrid streamer tori-line, long streamers were used for the first 80 m from the stern and a light streamer section followed in the next 70 m.

**Table 1:** Tori-line designs used in each experiment. In all tori-line, the first streamer was deployed 10 m astrn.

	Experiment 1	
	Light tori-line	Long tori-line
Line length	100 m	100 m
Line material	Polyester multifilament with nylon monofilament core	Nylon cord
Streamer number	80	16
Streamer length	1 m	7 m×4, 5 m×4, 3 m×4, 1 m×4
Streamer interval	1 m	5 m
Streamer material	PP band	Nylon cord

	Experiment 2		
	Light tori-line	Hybrid tori-line	Modified light tori-line
Line length	200 m	200 m	200 m
Line material	Polyester multifilament with nylon monofilament core		
Streamer number	80	85 (long streamer: 15, short streamer: 70)	90
Streamer length	0.5 m	long: 8.5, 8.0∼2.0, 1.5 m, short: 0.5 m	5.0 m×1, 0.5 m×4, ∼1.5 m×1, 0.5 m×4, 0.5×45
Streamer interval	1 m	long: 5 m, short: 1 m	1 m
Streamer material	PP band	long: UV-coated rubber tube, short: PP band	PP band

In order to maximize the aerial extent, we dragged a squid lure at the end of the tori-lines in experiment 1 and PP band to the in-water extent of the backbone in experiment 2. Four 0.25 m PP bands were spliced at 5 m intervals along the last 50 m of backbone in experiment 2.

### Data analyses

#### Experiment 1

The number of bycatch of Laysan albatross and black-footed albatross were estimated for each tori-line type. Generalized linear model (GLM) was used to analyze the effects of tori-lines on the bycatch number. The bycatch number was set as a response variable. Tori-line type (*TL*) and the vessel's ID (*VI*) were set as categorical variables and number of hooks used in each fishing operation (*HN*) is assumed as an offset variable. Because the number is countable data, we assumed that the bycatch number (*μ*_b1_) has a negative binomial distribution with two parameters (*α*, *β*),
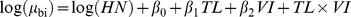
where *β*_0_–*β*_2_ are the estimated parameters of interest. We used the glm.nb function (MASS library, [19]) of R version 2.11.1 [20] to fit GLM. We used the likelihood-ratio test to compare the differences in the number of bycatch between tori-line types with and without *TL*.

#### Experiment 2

A hierarchical approach was used to compare the magnitude and distribution of seabird attacks among the three tori-lines. The mean number of attacks across the seven distance bins was compared during tori-line types or species types (albatrosses and shearwaters) using Fisher's exact test for species types.

Number of primary attack of Laysan albatross was calculated for each tori-line type. Generalized linear mixed model (GLMM) was used to analyze tori-line effects on the number of primary attack. The number was set as the response variable. Tori-line types (*TL*) and the order of operation block (*OP*) were set as categorical variables and abundance of Laysan albatross in each observation session (*LA*) was assumed as an offset variable. Because the number is countable data, we assumed that the number of attack (*μ*_a_) has a Poisson distribution.
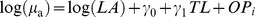
where *γ*_0_–*γ*_1_ are the estimated parameters of interest. The *OP_i_* is the random effect. We used the lmer function (lme4 library, [21]) of R version 2.11.1 to fit GLMM. We used the likelihood-ratio test to compare the differences in the number of attacks among tori-line types with and without *TL*.

## Results

### Experiment 1

**Figure 3:**
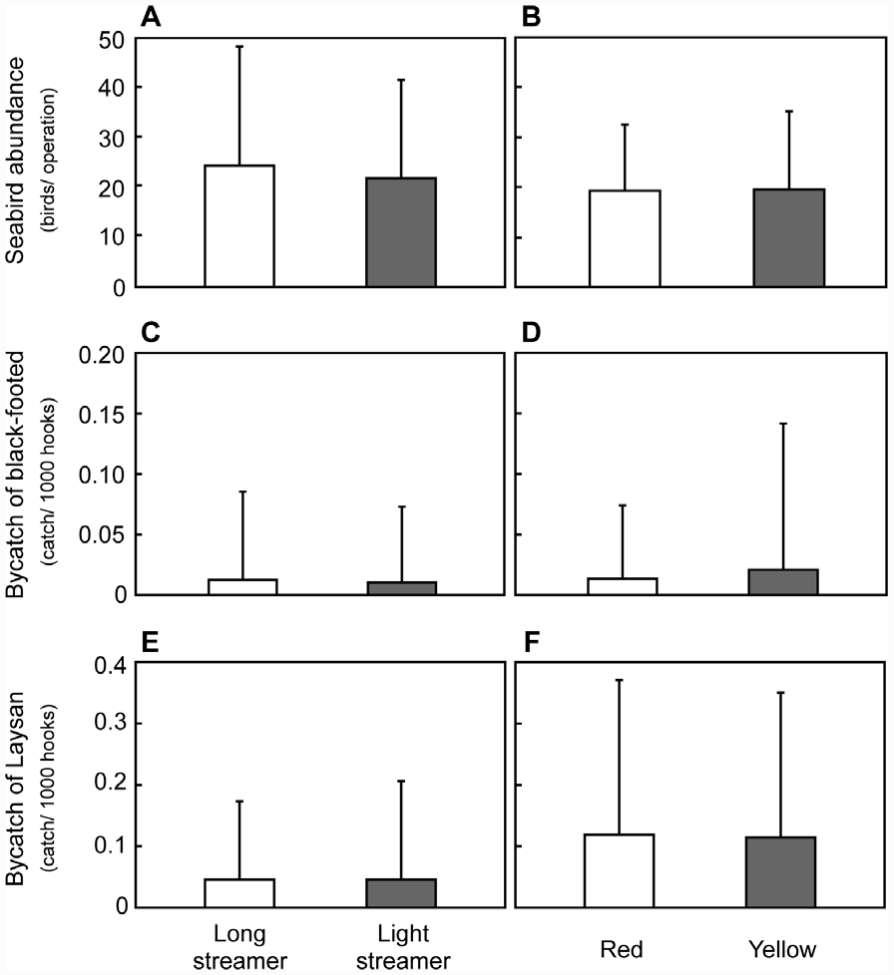
Comparison of tori-line types (left), and streamer colors of light streamer tori-lines (right) in experiment 1. Top, middle, and bottom rows shows seabird abundance (A and B), black-footed albatross bycatch (C and D), and Laysan albatross bycatch (E and F), respectively. Each vertical bar shows standard deviation.

In experiment 1, a total of 567 sets (2,130,570 hooks) was conducted and collected information. The seabird bycatch occurred only in Laysan albatross and black-footed albatross and the total bycatch numbers were 124 and 27, respectively. The number of seabirds observed appearing for each tori-line was not statistically different (Mann-Whitney *U*-test: tori-lines types; *U* = 18,766, *p* = 0.14; color types; *U* = 3716, *p* = 0.94, Figure 3A, B). There were no significant differences in the bycatch number between the tori-line types (GLM; black-footed albatross: *χ* ^2^ = 1.329, df = 1, *p* = 0.25, Figure 3C; Laysan albatross: *χ* ^2^ = 2.55, df = 1, *p* = 0.81, Figure 3E), and color types (GLM; black-footed albatross: *χ* ^2^ = 0.36, df = 1, *p* = 0.55, Figure 3D; Laysan albatross: *χ* ^2^ = 0.07, df = 1, *p* = 0.79, Figure 3F). On the other hand, there was significant difference in the bycatch number of long streamer vessels between phases 1 and 2 (Mann-Whitney *U*-test; *U* = 12256.5, *p*<0.01).

### Experiment 2

The light streamer and the modified light streamer tori-lines were trailed freely in the wind. On the other hand, the end part of long streamer in hybrid streamer tori-line was weighted so that remained in the seawater, and the streamer kept hanging and did not trail even when the wind blew strongly.

Data from 24 sets was obtained in this experiment. The mean number of seabirds observed appearing for each tori-line was 27.28±28.13 (mean ± SD), and albatrosses and shearwaters were 11.16±12.37 and 7.57±14.95, respectively. The proportion of albatrosses of all appearing seabirds were 40.9% (Laysan albatross: 37.9%; black-footed albatross: 2.9%), and Laysan albatross was the main seabird species that followed the vessel during line setting. While the appearance rates of shearwaters (e.g. northern fulmar (*Fulmarus glacialis*), short-tailed shearwater and wedge-tailed shearwater) and other birds (e.g. various species of gull (*Larus* spp.) and storm-petrel (*Oceanodroma* spp.) were 27.7% and 31.4%, respectively. However, most shearwaters and other birds were resting on the sea or passing, and did not follow the vessel during line setting. The number of seabirds observed appearing for each tori-line was not statistically different (Kruskal-Wallis test: albatross: *χ* ^2^ = 5.99, df = 2, *p* = 0.74; Laysan albatross: *χ* ^2^ = 5.99, df = 2, *p* = 0.66; shearwater: *χ* ^2^ = 5.99, df = 2, *p* = 0.93). For all tori-line designs, seabirds did not pass under the tori-line. Especially in some cases of using the modified light streamer tori-line, it seemed that seabirds were surprised by the motion of long streamers because they straightened up and turned quickly when they approached the tori-line.

**Figure 4:**
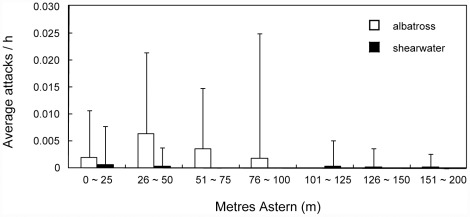
The difference of the primary attack distribution during seabirds in experiment 2. The distribution during albatrosses and shearwaters are represented as a function of distance astern to 200 m. Each vertical bar shows standard deviation.

**Figure 5:**
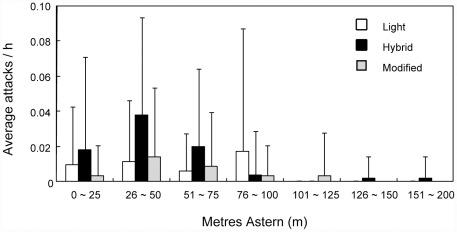
The difference of the primary attack distribution among three tori-line designs in experiment 2. The distribution of three tori line designs (Light, Hybrid and Modified) are represented as a function of distance astern to 200 m. Each vertical bar shows standard deviation.

A total of 88 primary attacks were recorded and 81% and 7% of them were carried out by albatrosses (Laysan albatross: 70 attacks; black-footed albatross: 1 attack) and shearwaters (northern fulmar (*Fulmarus glacialis*): 3 attacks; short-tailed shearwater: 1 attack; wedge-tailed shearwater: 2 attacks), respectively. Unidentified species of gull, lesser frigatebird (*Fregata ariel*), pomarine skua (*Stercorarius pomarinus*) attacked 6, 4 and 1 times, respectively. All attacks occurred on the starboard side of the tori-line. The number of attacks by albatrosses per fishing operation was more than 10 times higher than shearwaters. Overall the mean attack number of albatrosses (0.033 attacks per min) was also an order of magnitude higher than that for shearwaters (0.003 attacks per min). The primary attack number across the area monitored, the difference in the distribution of attack numbers was statistically significant between albatrosses and shearwaters (Fisher's exact test: *p*<0.01, Figure 4), and albatrosses seemed to attack within 100 m of the stern of the vessel. The difference among tori-line types was also not significant (Fisher's exact test: *p* = 0.35, Figure 5). In the primary attack number of Laysan albatross which attacked most aggressively of all seabirds, there was no significant difference among the tori-line types (GLMM: *χ* ^2^ = 4.88, df = 2, *p* = 0.09).

One third of primary attacks (31 times) led to a secondary attack, and 28 of the secondary attacks resulted from primary attacks made by Laysan albatross. The other secondary attacks resulted from primary attacks made by unidentified species of gull (1 time) and northern fulmar (2 times). One hundred and sixty five birds took part in the secondary attack, and 158 of them were Laysan albatrosses (the mean number = 5.3).

Total number of Laysan albatross caught was two, three and four, and the bycatch number was estimated at 0.011, 0.017 and 0.022 birds/1000 hooks for the light streamer tori-line, the hybrid streamer tori-line and the modified light streamer tori-line trials, respectively. Statistical tests were not conducted because the sample sizes were too small to test for any effect. On the other hand, no shearwaters were caught in this experiment.

## Discussion

The result in experiment 1 suggested that the bycatch of albatrosses is not significantly different between the long streamer and the light streamer tori-lines. The results in experiment 2 also suggested that the effectiveness for reducing the seabird attacks is not significantly different among the light streamer, the hybrid streamer and the modified light streamer tori-line. These results would suggest that the light streamer tori-line has the same effect in reducing bycatch of seabirds as the long streamer tori-line in the tuna longline fishery operating in the North Pacific.

The streamer length also did not affect the extent of the avoidance of seabird bycatch in both experiments. Seabirds did not pass under the tori-line even if it was a light streamer tori-line, which suggests that the long streamer did not more effectively guard the bait from being attacked by seabirds. The data collected by observers in the southern bluefin tuna (*Thunnus maccoyii*) fishery showed that long streamer tori-lines have similar effectiveness for mitigating seabird bycatch to light streamer tori-lines [22]. Seabirds may be surprised by the movement of the streamer. We could observe that seabirds were intimidated by the trailing long streamer of the modified streamer tori-line. Melvin et al. [15] has suggested that the working of tori-lines is not preventing attacks but rather displacing the seabirds further away from the vessel. They also noted that the ability of the hybrid streamer was higher than that of the light streamer. In their study, the end part of the hybrid long streamer might not have attached in the seawater, so the streamer might have trailed. It is considered that the heavy material of the hybrid long streamer (UV-coated rubber tube) showed complex movements. In future studies, it is necessary to examine the effectiveness of the streamer motion.

The color of streamer did not affect the bycatch in experiment 1. There is little information about which colors are effective as a detergent for minimizing attacks on baits. Although conspicuous colors may have a higher ability than somber color streamer, there may not be a difference of the ability to intimidate approaching birds among conspicuous colors such as red and yellow.

The bycatch in phase 2 was higher than in phase 1 in experiment 1. Even vessels which did not catch seabirds in phase 1, caught seabirds in phase 2. Environmental effects might relate with the difference of bycatch. The bycatch number has been shown to increase with the wind speed [8]. Strong winds may cause greater turbulence that keeps the bait nearer the surface of the sea for longer and also seabirds may find it easier to fly well in windier conditions making it easier to locate baits. Brothers et al. [8] has also suggested that the direction of the wind is related to the bycatch rate. When the wind was to the stern during setting, the thrown bait might get blown, and land on the area where the bait was not guarded by the tori-line.

In experiment2, although the appearance rate of Laysan albatross was relatively higher than other seabird during line settings, many shearwaters were also distributed in these operating areas. Nevertheless, most shearwaters did not follow the vessel. The most primary attacks were carried out by Laysan albatross. Laysan albatrosses were the main seabird which attacked the bait from April to June. All seabirds caught in experiment 1 were only albatrosses and no shearwaters were caught. Recent research conducted using a commercial pelagic longliner during 6 December 2010–10 January 2011 in the western North Pacific, showed that 90% of observed seabirds were Laysan albatrosses and most attacks were carried out by this species (Sato et al. unpubl.). This observation would suggest that the albatross bycatch in experiment 1 was mainly due to primary attacks by Laysan albatrosses not by a secondary attack on bait brought to the surface by shearwaters. Moreover, in tuna longline research from 1992 to 2005 conducted throughout the year by Japanese training and research vessels in North Pacific, 98% of the all bycatch seabirds were albatrosses [23]. From these results, it is considered that albatrosses, especially Laysan albatross, are the main seabird which attack longline baits in the western North Pacific.

There were few primary attacks by shearwaters and no shearwaters were caught in this study. These results differ from the results that diving seabirds such as white-chinned petrel (*Procellaria aequinoctialis*) and Cape gannet (*Morus capensis*) aggressively attacked the bait and were incidentally caught, and also carried out many secondary attacks in the South African EEZ [15]. Attacks by shearwaters on the deployed bait were rather lower in the North Pacific than off South Africa. On the other hand, the distribution of albatross's primary attacks in this study was similar with that in the South African EEZ [15], and most attacks were conducted within 100 m of the stern. In both studies, attack numbers per 1000 hooks were around 0.04 and it seems that there are no large differences in bycatch rates. In contrast with observations on shearwaters, the aggression of albatrosses to baited hooks in the North Pacific was similar to that in the South African EEZ.

In conclusion, light streamer tori-lines were as effective as tori-lines with long streamers for avoiding seabird bycatch in the North Pacific. This result may be related with seabird characteristics which is the low aggression of the diving seabirds in the North Pacific. During conversations with skippers who participated in this study, many skippers reported that long streamer tori-lines caused much trouble due to tangling. Light streamer tori-lines did not caused much trouble due to tangling. This merit would further encourage fishermen, who are obliged to use mitigation methods as a part of the WCPFC regulations, to use tori-lines.
